# Tumorigenicity and Validity of Fluorescence Labelled Mesenchymal and Epithelial Human Oral Cancer Cell Lines in Nude Mice

**DOI:** 10.1155/2016/4897986

**Published:** 2016-11-13

**Authors:** Wei Xin Cai, Li Wu Zheng, Li Ma, Hong Zhang Huang, Ru Qing Yu, Roger A. Zwahlen

**Affiliations:** ^1^Department of Oral and Maxillofacial Surgery, Guanghua School of Stomatology, Hospital of Stomatology, Guangdong Provincial Key Laboratory of Stomatology, Sun Yat-sen University, Guangzhou, China; ^2^Discipline of Oral Diagnosis and Polyclinics, Faculty of Dentistry, The University of Hong Kong, Pokfulam, Hong Kong; ^3^Discipline of Oral Rehabilitation, Faculty of Dentistry, The University of Hong Kong, Pokfulam, Hong Kong; ^4^Discipline of Oral and Maxillofacial Surgery, Faculty of Dentistry, The University of Hong Kong, Pokfulam, Hong Kong

## Abstract

Tumorigenicity and metastatic activity can be visually monitored in cancer cells that were labelled with stable fluorescence. The aim was to establish and validate local and distant spread of subcutaneously previously injected fluorescence transduced human tongue cancer cell lines of epithelial and mesenchymal phenotype in nude mice. A total of 32 four-week-old male athymic Balb/c nude mice were randomly allocated into 4 groups (*n* = 8). A single dose of 0.3 mL PBS containing 1 × 107 of four different cancer cell-lines (UM1, UM1-GFP, UM2, and UM2-RFP) was injected subcutaneously into the right side of their posterolateral back. Validity assessment of the labelled cancer cells' tumorigenicity was assessed by physical examination, imaging, and histology four weeks after the injection. The tumor take rate of cancer cells was similar in animals injected with either parental or transduced cancer cells. Transduced cancer cells in mice were easily detectable in vivo and after cryosection using fluorescent imaging. UM1 cells showed increased tumor take rate and mean tumor volume, presenting with disorganized histopathological patterns. Fluorescence labelled epithelial and mesenchymal human tongue cancer cell lines do not change in tumorigenicity or cell phenotype after injection in vivo.

## 1. Introduction

A fluorescent labelling of human oral cancer cell lines, stable over several generations, might provide the possibility to visually monitor their tumorigenicity and metastatic activity in vivo.

Both green fluorescent protein (GFP) and red fluorescent protein (RFP) (GenTarget, Inc., San Diego, USA) have been extensively used to study cancer biology in animal models due to their stability after transduction [1]. As transduced cancer cells and their daughter cells persistently expressed the fluorescent proteins, a direct long-term visualization in vivo became facilitated [2–4].

In mice GFP/RFP labelled murine cancer cell lines were used to study the role of epithelial mesenchymal transition (EMT) in distant metastasis; it was detected that cells of epithelial and mesenchymal phenotype showed a fair share in the formation of distant metastasis [5]. The same authors, however, did not comment on an earlier report regarding negative effects of GFP transduction onto long-term development of colon cancer micro- and macrometastases in mice [1].

Cancer metastasis and local cancer invasion are considered to be closely related to specific cell phenotypes and their dynamic transition [6–8]. A negative in vitro interaction between GFP/RFP transduction and epithelial and/or mesenchymal phenotypes of human tongue cancer cell lines UM1 and UM2 has already been excluded previously, where it was detected that transduced UM1-GFP and UM2-RFP human tongue cancer cell lines maintained their particular original cell phenotype (Cai et al. in press).

Still, verification is needed, if those in vitro findings for transduced human cancer cells mentioned above also prevail in vivo in hairless “nude” mice. This particular animal model is appropriate (1) for subcutaneous tumor implantation and its subsequent clinical assessment [9] and (2) to detect fluorescence expressing cancer cells in living animals by means of specific intravital imaging techniques [10].

This study aimed to establish and validate a novel in vivo nude mice model where subcutaneously injected transduced human tongue cancer cell lines of epithelial and mesenchymal phenotype could be visualized under a fluorescent microscope.

## 2. Materials and Methods

### 2.1. Cell Culture

The GFP/RFP transduction procedure and cell culture conditions were described in our previous publication (Cai et al. in press). In brief, two human tongue cancer cell lines UM1 and UM2 (donation from Dr. David Wong, School of Dentistry, University of California Los Angeles, USA) were cultured in an incubator at 37°C/5% CO_2_ using DMEM/F-12 medium (DMEM/F-12, a mixture of Dulbecco's Modified Eagle Medium and Ham's F-12 medium at 1 : 1, Sigma, New York, NY, USA). UM1 and UM2 demonstrated mesenchymal and epithelial cell features, respectively. GFP and RFP lentiviral particles stock (GenTarget, Inc., San Diego, America) was added into the confluent cells and maintained for 72 hours. The transduced cells were then selected for two weeks using G418 (Sigma, New York, NY, USA). The transduction efficiency and stability of the transduced cells were then assessed qualitatively and quantitatively.

### 2.2. Animal Care

The animal study was approved by the Committee on Use Live Animal for Teaching and Research (CULATR 3088-13), The University of Hong Kong. The experimental animal, male athymic Balb/c nude mice (Charles River Lab, Wilmington, USA) were kept in the Laboratory Animal Unit, The University of Hong Kong. Veterinarians from the laboratory animal unit fed all mice with standard rodent diet (LabDiet, St. Louis, USA) and autoclaved water ad libitum and checked their general state. The feeding room was maintained on a 12 : 12 hour light-dark circle at room temperature, 16–22°C. All animals were sacrificed 4 weeks after cancer cell injection.

### 2.3. Xenograft of Cancer Cells

The cancer cell xenograft, beforehand randomly allocated into 4 groups, was carried out on four-week-old mice. Animals in group A, group B, group C, and group D were injected with UM1, UM1-GFP, UM2, and UM2-RFP cells, respectively (Table 1). A single dose of 0.3 mL PBS containing 1 × 107 cancer cells was harvested and injected subcutaneously at the inferior right dorsolateral site into the mice immediately after the harvest.

### 2.4. Assessment of Tumorigenicity and Validity

The tumorigenicity and validity of the fluorescence labelled cancer cells in nude mice were assessed by physical examination, imaging, and histologic examination.

Emerging xenograft tumors were physically examined, measured in length and width, and recorded by the same investigator (CWX). The tumor volume was calculated for depicting a growth curve. A standard equation [11] was used for the volume calculation: tumor volume = length × width^2^ × 0.52.

The tumor take rate describes the percentage of mice that develops a palpable tumor until the sacrifice time.

The fluorescence-positive tumors of the mice injected with transduced cancer cells in groups B and D were observed under the in vivo imaging system (IVIS) spectrum (Perkin Elmer Inc., Waltham, USA) after being sacrificed by intraperitoneal injection of 150 mg/kg pentobarbital sodium (Alfasan Diergeneesmiddelen BV, Woerden, Netherlands). Tumor tissue and lungs of the mice in groups B and D were harvested and examined. The results were recorded and analysed using the Living Image 4.4 software (Perkin Elmer Inc., Waltham, USA).

Fresh, unfixed lung and tumor tissue of groups B and D animals underwent cryosection, to detect directly fluorescence labelled cancer cells. The specimens were immersed in a 2 : 1 mixture of 20% sucrose (Electron Microscopy Science EMS, Hatfield, USA) and Tissue-Tek® optimum cutting temperature (OCT) compound (EMS, Hatfield, USA) for 30 minutes, followed by another 30 minutes immersion in a 1 : 1 mixture of 20% sucrose and Tissue-Tek OCT compound. Thereafter the specimens were embedded in Tissue-Tek OCT compound in a tissue mold and frozen (EMS, Hatfield, USA). The frozen specimens then were transferred onto a metal grid and cut into tissue slices of 6 *μ*m thickness in a cryostat, to be finally observed under a fluorescence microscope (Nikon, Tokyo, Japan). Harvested tumor tissue and lungs from mice of groups A and C were fixed in 10% neutral formalin solution and infiltrated in paraffin using Shandon Excelsior ES® Operator (Thermo Fisher Scientific Inc., Waltham, MA, USA). The fixed tissue was cut with a microtome (Leica RM2155; Nussloch, Germany) into 6 *μ*m thick slices and histologically examined under a light microscope (Leica DMLB, Nussloch, Germany), after being stained with hematoxylin and eosin (H&E).

### 2.5. Statistical Analysis

The SPSS version 20 (Chicago, IL, USA) was used for statistical analyses. A nonparametric chi square test was applied to compare the tumor take rate between the different groups. The independent *t*-test was used to compare the mean tumor volume among the different groups. Differences between groups have been considered to be significant when *p* ≤ 0.05.

## 3. Results

### 3.1. Physical Examination

**Figure 1:**
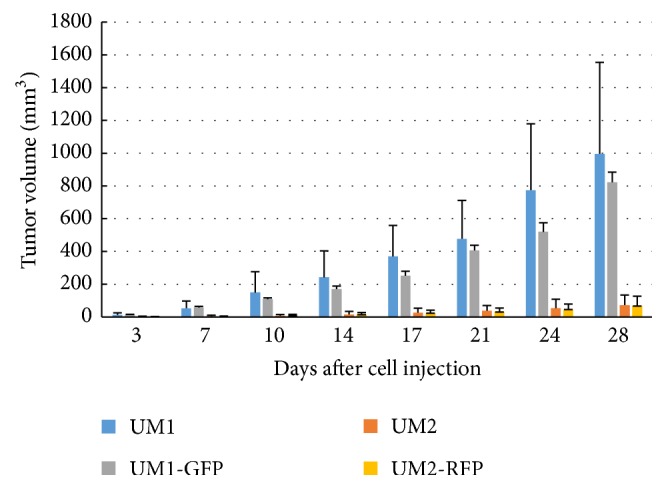
Tumor growth. The volume of the subcutaneous tumor was measured at 8 time points (3, 7, 10, 14, 17, 21, 24, and 28 days after cell injection) during the experiment. The mean tumor volume and the standard deviation for all four groups are shown.

**Figure 2:**
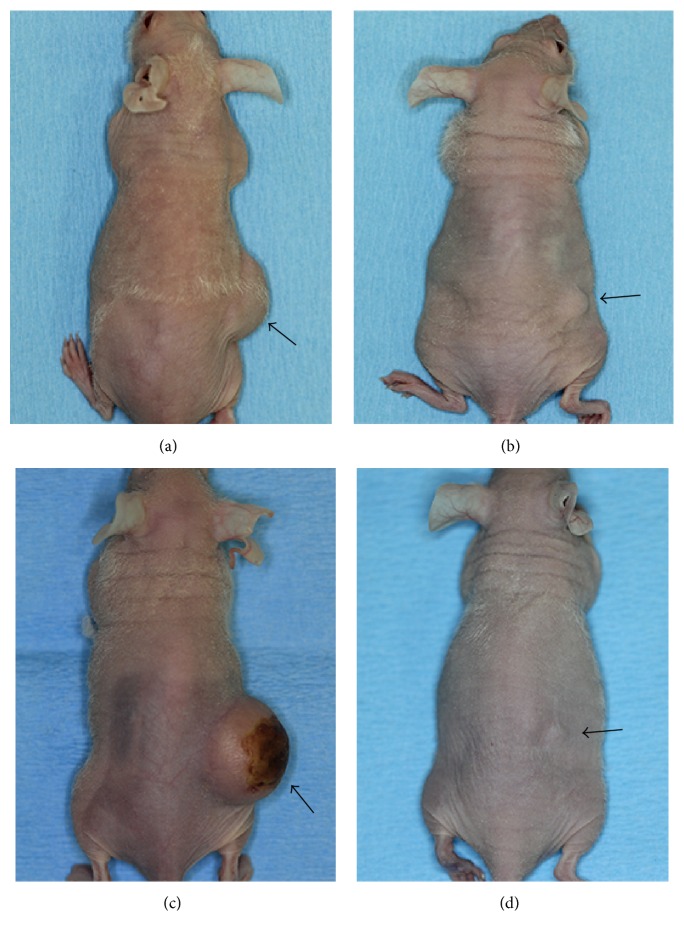
Subcutaneous tumor at injection site. Physically palpable tumor masses are shown which formed subcutaneously at the injection site. The injected cells in each group (a) UM1; (b) UM2; (c) UM1-GFP; (d) UM2-RFP.

The subcutaneously injected cancer cells thrived (Figure 1) and became physically palpable tumors at the right posterior dorsolateral injection sites (Figure 2). The tumor take rate of cancer cells and the tumor volume of all four groups are summarized in Tables 2 and 3, respectively. The transduced cancer cell lines showed a similar tumor take rate compared to their parental cancer cells (*p* = 0.302 for UM1, *p* = 0.615 for UM2). The tumor volume between parental and transduced cell lines was not significantly different (*p* = 0.501 for UM1, *p* = 0.314 for UM2). The tumor take rate (*p* = 0.021) and the tumor volume (*p* = 0.003) emerged to be statistically significant between the parental cell lines UM1 and UM2. Between the transduced daughter cell lines UM1-GFP and UM2-RFP on the contrary, the tumor take rate did not differ significantly (*p* = 0.248), whereas the tumor volume differ highly significantly (*p* = 0.002).

### 3.2. In Vivo and Ex Vivo Fluorescent Imaging

**Figure 3:**
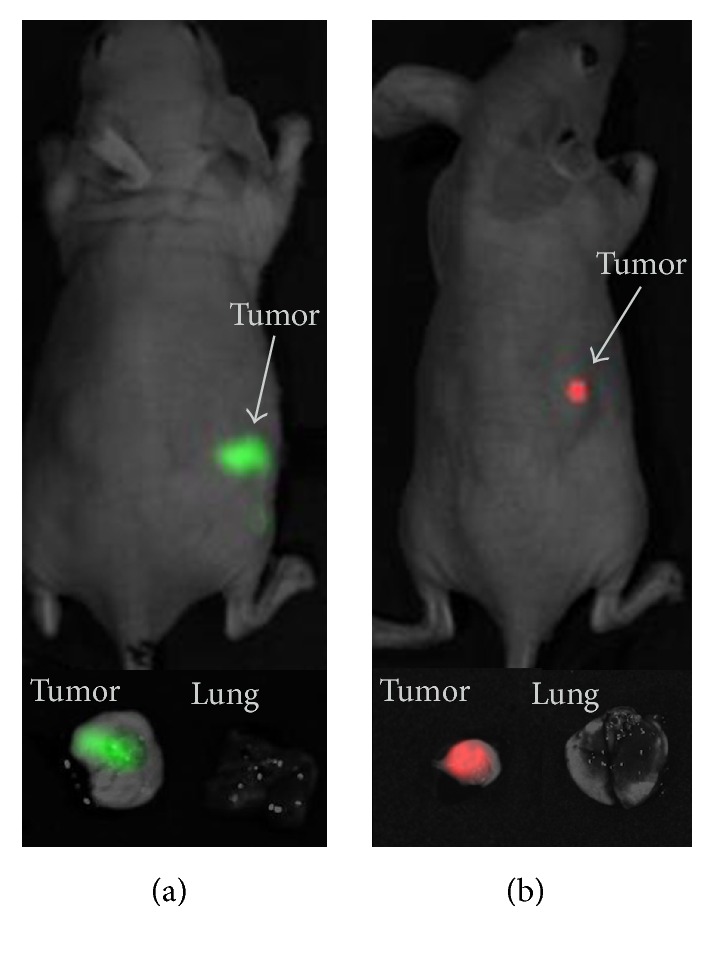
In vivo and ex vivo fluorescent imaging of tumors and lung specimens. The inoculated tumors derived from UM1-GFP (a) and UM2-RFP (b). Whereas the upper part of the image highlights the fluorescence in vivo, fluorescence of harvested tumor tissue and lung specimens is shown in the lower part of the image.

Due to their stable and intense signal transduced cancer cell lines could be easily detected in the animals under fluorescent imaging (Figure 3). In mice of groups B (UM1-GFP inoculation) and D (UM2-RFP inoculation) ex vivo fluorescent imaging detected fluorescent signals in tumors, however, not in lung tissue (Figure 3).

### 3.3. Histology

**Figure 4:**
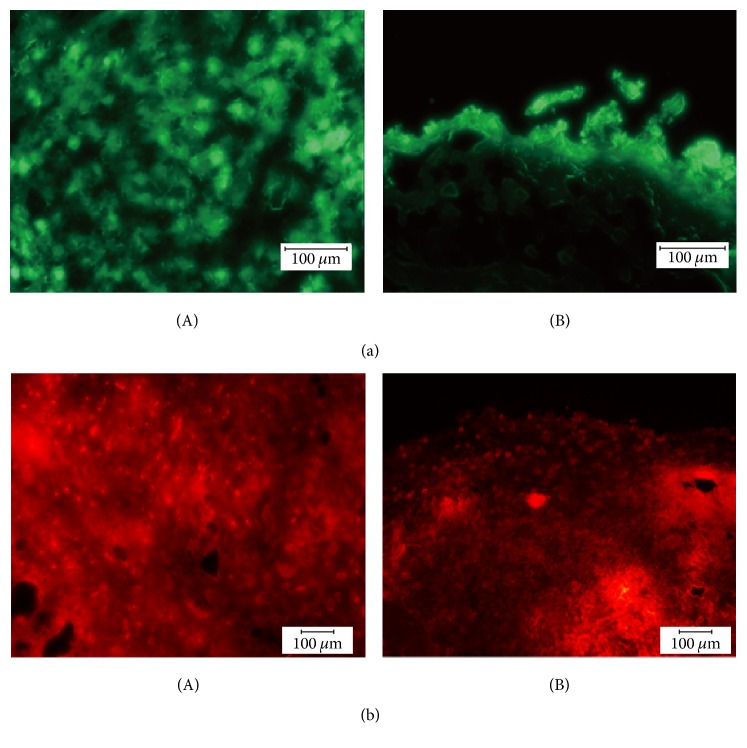
(a) Cryosection of a UM1-GFP tumor. The tumor presents a disorganized cell pattern (A) (magnification 40x) with an aggressive invasion front (B) (magnification 20x). (b) Cryosection of a UM2-RFP tumor. A clear border (B) (magnification 20x) between well-defined tumor cells (A) (magnification 40x) and the surrounding can be detected.

The cryosections of fresh tumor tissue harvested from groups B and D animals displayed clear signals under the fluorescent microscope (Nikon TIRF microscope, Nikon, Tokyo, Japan). Their histological properties could be observed by fluorescent imaging obtained from cryosections. Whereas tumors of the UM1-GFP cell lines displayed a disorganized tissue structure with an aggressive invasion front toward the surroundings (Figure 4(a)), tumors of the UM2-RFP cell lines presented a well-defined mass with clear borders (Figure 4(b)). Lung tissue of those mice exhibited no fluorescent signals.

**Figure 5:**
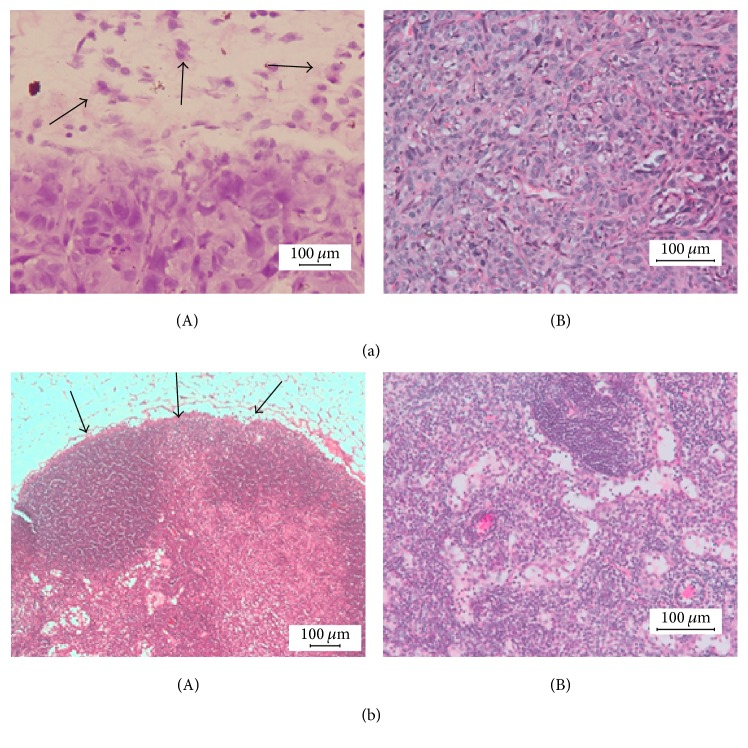
(a) H&E staining of a UM1 tumor. (A) An invasive cancer pattern is shown at the front (black arrows, magnification 100x) of UM1 derived cancer tissue. (B) Poor cell differentiation is observed within the tumor specimen (magnification 200x). (b) H&E staining of a UM2 tumor. (A) A well-defined border to the surrounding tissue is visible (black arrows, magnification 100x). (b) The cancer tissue is well differentiated (magnification 200x).

H&E stained UM1 cell line specimens showed poorly differentiated tumor tissue with invasive growth patterns (Figure 5(a)), while tumors of UM2 cell lines displayed well differentiated features with well-defined cancer borders (Figure 5(b)). Metastatic foci in H&E stained lung tissue could not be found in any animal of whatsoever group.

## 4. Discussion

In vivo tumorigenicity of two subcutaneously injected fluorescence labelled human tongue cancer cell lines of epithelial and mesenchymal phenotype was verified and validated by means of histopathologic examination and both in vitro and in vivo imaging.

Nonprogress or even regression of xenografted cancer represent an infringement of cancer cells in animal models [12]. This experiment revealed a high tumor take rate (87.5% for UM1-GFP, 62.5% for UM2-RFP) of transduced human tongue cancer cell lines, whose fluorescent signals remained stable in vivo until the expected time of euthanasia. Growth patterns of fluorescent tumors were similar to those of the parental, wild type, cell lines. Therefore, both similar tumor take rate and volume between parental and transduced cell lines verify the extant tumorigenicity of transduced human tongue cancer cell lines. Moreover, histopathologic properties of fluorescent cell lines were found to be similar to those of the wild type cell lines and sustain together with the tumorigenicity their validity in nude mice.

It is advocated that transformation from the epithelial into the mesenchymal phenotype of cancer cells is important both for local invasion and distant metastasis [8, 13–16]. The mesenchymal phenotype of the UM1 cell line has already been investigated and proven in vitro [17]; physical and histologic findings of the here presented study point at its promoting role in in vivo tumor formation and development.

The UM1 cell line presented (1) a higher tumor take rate with an increased mean tumor volume and (2) a propagated disorganization of its histopathologic growth pattern, a correlation having been suggested before [18, 19]. Fluorescent imaging of the UM1-GFP cell line tumor further depicted its invasive tumor front, supporting the validity of the transduced UM1 cancer cell line in this study model.

The lung was designated as the metastatic target organ in the present study, because it was reported to be with 70% the most frequently involved metastatic site [20]. The negative result of lung metastases in this study, however, has to be considered nonpersuasive because other potential organs such as liver, brain, and bone have not been tested. Labelling with two different colors provided an easy differentiation between cancer cells of different phenotypes. Subcutaneous injection of fluorescence labelled cancer cells in nude mice is an established technique in cell cancer biology studies [21]. In this experimental setup, however, the inherent heterogeneity between subcutaneous space and tongue tissue might represent a shortcoming. Future experiments with these cell lines might benefit therefore from injection into the tongue and hence from an orthotopic tumor model which probably would be more persuasive.

A long-lasting fluorescent expression allows direct and timely less limited imaging possibilities of in vivo cancer development. Noninvasive whole-body imaging therefore represents an appropriate monitoring of cancer growth and progression [22]. Simultaneous application of GFP and RFP in one animal is feasible due to distinct light spectra between both colors [23]. Since cancer cells of epithelial or mesenchymal phenotypes are long lastingly labelled with distinct fluorescence, it may become feasible in the future to simultaneously investigate cancer progression such as local invasion and distant metastasis using both cell types in a single animal model.

## 5. Conclusions

Long-lasting fluorescence labelling of epithelial and mesenchymal phenotype human tongue cancer cell lines does not change neither tumorigenicity nor phenotype in vivo and allows continuous monitoring by noninvasive in vivo visualization.

## Figures and Tables

**Table 1:** Xenograft of parental and transduced tongue cancer cells.

	Animals	Age (weeks)	Cells	Sacrifice time
A	8	4	UM1	Week 4
B	8	4	UM1-GFP	Week 4
C	8	4	UM2	Week 4
D	8	4	UM2-RFP	Week 4

**Table 2:** Tumor take rate among the four groups (mean ± SD, %).

Group	UM1	UM2	*p* value

Parental	100 (8/8)	50 (4/8)	0.021
Transduced	87.5 (7/8)	62.5 (5/8)	0.248
*p* value	0.302	0.615	—

**Table 3:** Tumor volume among the four groups (mean ± SD, mm^3^).

Group	UM1	UM2	*p* value
Parental	995.8 ± 558.4	72.4 ± 61.3	0.003
Transduced	822.5 ± 379.2	67.5 ± 59.0	0.002
*p* value	0.501	0.314	—
